# Retraction: Primary extraosseous Ewing sarcoma of the lung in children

**DOI:** 10.3332/ecancer.2013.332

**Published:** 2013-07-15

**Authors:** Nidal Alsit, Clara Fernandez, Jean Luc Michel, Linda Sakhri, Audrey Derouet, Augustin Pirvu

**Affiliations:** 1 Department of Thoracic, Vascular, and Cardiac Surgery, University Hospital Felix Guyon, Reunion, France; 2 Department of Pathological Anatomy, University Hospital Felix Guyon, Reunion, France; 3 Department of Pediatrics Surgery, University Hospital Felix Guyon, Reunion, France; 4 Department of Onco-pneumology, University Hospital Grenoble, France; 5 Department of Pediatrics, University Hospital Felix Guyon, Reunion, France; 6 Department of Thoracic, Vascular, and Endocrine Surgery, University Hospital Grenoble, France

## Abstract

*e*cancer **7** 312 (2013)

The report in this article of the treatment the patient received is incorrect. Three of the authors (Jean Luc Michel, Clara Fernandez and Audrey Derouet) were unaware that their names had been added to the author list. The three remaining authors (Nidal Alsit, Linda Sakhri and Augustin Pirvu) were not involved with the treatment of the patient.

